# Modifying recombinant purple acid phosphatase using computational design

**DOI:** 10.1007/s00249-025-01779-3

**Published:** 2025-07-12

**Authors:** Aishwarya Venkatramani, Montader Ali, Olga Predeina, Jennifer C. Molloy, Pietro Sormanni, Elizabeth A. H. Hall

**Affiliations:** 1 Department of Chemical Engineering and Biotechnology, University of Cambridge, Cambridge, CB3 0AS UK; 2 Yusuf Hamied Department of Chemistry, University of Cambridge, Cambridge, UK

**Keywords:** Purple acid phosphatase, Protein engineering, Thermal stability, Computational design

## Abstract

Enhancing protein stability while maintaining activity is a long-standing challenge in protein engineering, as modifications that benefit one property often compromise another. In this study, we leveraged a computational design strategy, CamSol Combination, to make a first step to improve the stability of purple acid phosphatase (PAP), a metalloprotein known for its distinctive pink color. PAP serves as a challenging model for engineering due to its complex redox-active site and the incorporation of iron ions critical to its function. Five mutations were introduced—H22R, A24P, F54P, H197P, and T208R—targeted to enhance thermal stability, as suggested by the computational design pipeline, while avoiding key functional regions. Experimental validation confirmed the choice of mutations with a 5 °C increase in thermal stability and retained enzymatic activity across a slightly expanded pH range. The mutations introduced subtle shifts in the enzyme’s spectral and redox behavior, consistent with a lower energy of the oxidized state, and with dynamic light scattering data suggesting low aggregation. These results highlight the potential of computational approaches like the CamSol Combination to streamline protein engineering by enabling multi-trait optimization.

**Supplementary Information:**

The online version contains supplementary material available at 10.1007/s00249-025-01779-3.

## Introduction

Optimizing the solubility and conformational stability of proteins, while preserving their biological activity, represents one of the critical, yet challenging frontiers in protein engineering, as these properties directly influence the expression levels and efficacy. Proteins frequently exhibit a delicate balance between stability, solubility, and biological function, where modifications improving one characteristic can inadvertently compromise another (Jain et al. 2017). This is especially pronounced in metalloenzymes like purple acid phosphatases (PAPs), which rely heavily on precise metal coordination and structural integrity for activity (Bhadouria and Giri 2022). PAPs hold considerable promise across diverse sectors, including agriculture, biotechnology, environmental bioremediation, and pharmaceuticals, due to their unique catalytic abilities and tolerance of harsh conditions (Sharma et al. 2023). In the medical field, elevated levels of PAPs have been associated with conditions such as osteoporosis and certain bone malignancies, making them targets for therapeutic interventions. As an acid phosphatase, PAP catalyzes the hydrolysis of phosphate monoesters within a pH range of 4–7 and has a distinguishing feature in its resistance to L-tartrate inhibition, which is why mammalian PAPs are often referred to as tartrate-resistant acid phosphatases (TRAPs) (Hayman et al. 2000; Andersson and Ek-Rylander, 1995; Reithmeier et al. 2017). In mammals, PAP exists as glycosylated monomers and plays crucial roles in metabolic processes, particularly in phosphate acquisition and utilization (Bhadouria et al. 2017; Bhadouria and Giri 2022). Given PAP's significance as a biochemical marker, developing a reliable method for its recombinant production is crucial for advancing research and clinical applications.

However, their practical implementation is often limited by issues of conformational instability, constraining their efficiency and applicability. Addressing these challenges through rational, computationally guided mutations could not only advance fundamental enzyme engineering, but also unlock the potential for PAPs to serve as robust, versatile catalysts in industrial and environmental applications, and as viable therapeutic targets in medical treatments.​

In this study, we explore the application of a computational design strategy, CamSol Combination, to enhance the stability of a mammalian purple acid phosphatase (PAP). The combination pipeline, previously employed in antibody design (Rosace et al. 2023), and alpha-amylase (Ali et al. 2024) studies, has demonstrated its ability to identify variants with improved stability and solubility. Advancements in computational approaches, such as the CamSol Combination pipeline, offer promising strategies to overcome such challenges related to protein expression and solubility. In the context of purple acid phosphatase (PAP), the goal was to identify and test a subset of multiple mutations that were predicted to improve stability and establish whether the protein’s functional integrity was maintained.

To achieve this, the CamSol combination pipeline, as described in detail by Rosace et al. (2023), was used to evaluate protein stability and solubility by targeting residues that may be part of an aggregation-promoting hotspot, or may have a stability liability, as suggested by their low frequency in a multiple-sequence alignment (MSA) of related proteins. CamSol also allows critical functional and glycosylation sites to be preserved, by excluding such sites from the list of possible mutations. This makes it a valuable tool for engineering challenging proteins like PAP. However, these computational tools primarily address solubility and stability issues and do not directly account for challenges associated with glycosylation or the redox environment in non-native expression systems. PAPs are distinguished by a heterovalent metal center, typically Fe (III)–Fe (II) in mammalian PAP, which is essential for catalytic activity (Merkx and Averill 1998). When the metal center is oxidized to Fe (III)–Fe (III), PAP transitions to a non-catalytic form. Thus, as a first step in this study, we aimed to identify mutations that improve PAP's stability in a mammalian expression system without unfavorable impact on substrate activity or redox behavior.

## Materials and methods

### Computational design rationale

Previous studies have demonstrated that a combination of mutations are often necessary to achieve substantial improvements in protein stability and solubility (Sormanni et al. 2015; Bhadouria et al. 2017; Bhadouria and Giri 2022). These findings show that, while a stepwise approach allows for the evaluation of individual amino acid substitutions and their impact on protein structure and activity, the effects of multiple mutations are not necessarily additive or dominated by a single mutation, which alone has a high positive or negative impact. For example, in the case of *Bacillus licheniformis* α-amylase (BLA), a variant incorporating nine mutations exhibited significant improvements in stability and solubility while retaining enzymatic activity and hyper-thermostability (Ali et al. 2024). This suggests the possibility of the additive effects of multiple mutations being greater than the effect of a single mutation and underscores the potential of computational tools, such as CamSol Combination, to guide the rational selection of multiple mutation sites for optimizing protein properties, particularly in complex enzymes like PAP.

### CamSol combination

Mutations were predicted using the CamSol Combination web server (www-cohsoftware.ch.cam.ac.uk—University of Cambridge, UK), which integrates four predictive components: (1) the CamSol intrinsic solubility profile (a predictive model that estimates the likelihood of a residue or mutation to promote solubility and reduce aggregation, based on both sequence and structure; a positive CamSol score suggests a solubilizing effect); (2) structure-corrected surface aggregation propensity; (3) a FoldX-based prediction of ΔΔ*G* for stability (ΔΔ*G* is the predicted change in folding Gibbs free energy upon mutation, calculated as Δ*G*_mutant – Δ*G*_wild type; a negative ΔΔ*G* indicates a thermodynamically stabilizing mutation); and (4) a position-specific scoring matrix (PSSM) derived from homologous sequences using HHBLITS. A cleaned version of the 1WAR PDB structure was used as input.

HHBLITS (toolkit.tuebingen.mpg.de/tools/hhblits—MPI Bioinformatics Toolkit) generated MSAs (.a3m format) were submitted using the MPI Bioinformatics Toolkit. Mutations were retained if they showed ΔΔ*G* < 0, positive log-likelihood in the PSSM, and increased CamSol score. The “alignment frequency strong filter” was disabled to allow potentially rare, but stabilizing mutations like proline to be considered. Residues directly coordinating the active site metals or involved in glycosylation were excluded from mutagenesis using the “Residues that can’t be changed” input (e.g., TYR53, HIS90, ASN95). Mutation combinations of up to eight were allowed, and chain similarity clustering enabled to maintain sequence diversity.

Candidate mutations were selected based on low predicted ΔΔ*G* values, which estimate their effect on stability and are calculated only for a subset of mutations that are evolutionarily grounded as inferred from an MSA of related proteins (Rosace et al. 2023). Meanwhile, intrinsic solubility scores were used to assess their potential impact on aggregation propensity. Since ΔΔ*G* primarily reflects the predicted stability improvement, it served as the primary metric for selection. While the webserver output prioritized designs by ΔCamSol and ΔΔ*G* scores, final mutation selection also involved manual inspection of residue positions, secondary structure elements, and potential impact on enzymatic function. To assess whether the selected mutations might induce significant structural perturbations, structural models of both wild-type and mutant PAP were generated using AlphaFold v2.3.1 with default parameters and full database configuration.

The final mutation set H22R, A24P, F54P, H197P, and T208R was selected for experimental validation.

### Plasmid and construct preparation

The purple acid phosphatase (PAP) sequence from PDB code *1WAR* (Sträter et al. 2005), optimized for mammalian expression, was synthesized with a CD33 signal peptide in the pcDNA3.4 vector, including a Kozak sequence and a C-terminal 3xFLAG and 6xHis tag. BsmBI (New England Biolabs, cat. R0739S) restriction sites were introduced to facilitate compatibility with the pcDNA3.4 vector (Thermo Fisher Scientific, cat. A14697), and the construct was assembled using Golden Gate Assembly. The resulting plasmid DNA was transformed into *E. coli* TOP10 cells (Thermo Fisher Scientific, cat. C404003), which were grown following the instructions of a standard midiprep kit. The plasmid was then purified using the Qiagen EndoFree plasmid midiprep kit.

### Cell transfection and protein expression

The purified plasmid was transformed into Expi293F cells (Thermo Fisher Scientific, cat. A14527) and cultured in Expi293 medium (Thermo Fisher Scientific, cat. A1435101). Cells were seeded to a final density of 3 × 10^6^ of viable cells/mL. Cells were transfected using ExpiFectamine 293 Reagent Kit (Thermo Fisher Scientific, cat. A14525) with plasmid DNA at a concentration of 1 µg/mL. After transfection, the cells were grown for 6 days, by which time sufficient protein expression levels were achieved for purification and initial investigation.

### Protein purification

To capture the His-tagged enzyme, AmMag™ Ni Magnetic Beads (GenScript, cat. L00776) were added to the media at a concentration of 0.2 ml per liter of cell culture. These were incubated at 4°C for 2 h, rolling at 200 r.p.m. Magnetic beads were collected, washed in PBS (Sigma-Aldrich, cat. P4417) and loaded onto the GenScript AmMag SA Plus purification system (GenScript, cat. L01013), where the beads were further washed. The beads were then washed with a 40mM imidazole buffer (Sigma-Aldrich, cat. I2399) and eluted in 10 mL of 200mM imidazole elution buffer. The eluted protein was concentrated and further purified using an ÄKTA purification system in a Superdex 75 Increase 10/300.

### UV–Vis and activity assay and oxidation/reduction of PAP

PAP activity was assayed in a 96-well Greiner polypropylene microplate using p-nitrophenyl phosphate (pNPP) disodium salt (Sigma Aldrich CAS 4264-83-9) as the substrate. Hydrolysis of pNPP by PAP produced p-nitrophenol (pNP), which has an absorbance peak at 410 nm. Measurements were taken over a pH range of 3.25–8.0 using various buffers: 0.1 M acetate (pH 3.5–5.5), 0.1 M MES buffer (Sigma-Aldrich, cat. M3671) (pH 6.5), 0.1 HEPES buffer (Sigma-Aldrich, cat. H3375) (pH 7.5) and 0.1 M Tris–HCl (Thermo Fisher Scientific, cat. 15504-020) (pH 8.5). Absorbance at 410 nm was recorded at 1-min intervals for 80 min at 22°C, 32°C, and 42°C using a CLARIOstar Plus plate reader (BMG LABTECH). Due to the pink color of PAP, a full UV–Vis absorbance spectrum was measured to characterize the protein’s absorbance properties. Both isolated wild-type and mutant PAP were oxidized with 0.5 µM of potassium peroxydisulfate in PBS. The absorbance peak for oxidized and isolated PAP was plotted and peak deconvolved using Origin Lab software (OriginLab Corporation, Northampton, MA, USA).

### Nano differential scanning fluorimetry

Protein samples were standardized to 1 µM in 50 µL of PBS buffer (pH 7.4). Thermal unfolding profiles were measured using a Nano-DSF (Prometheus NT.48, NanoTemper Technologies, Munich, Germany). The unfolding was monitored at a heating rate of 1°C/min from 20°C to 90°C in 10 µL glass capillaries. The unfolding temperature was detected by monitoring temperature-induced changes in the ratio of tryptophan fluorescence at emission wavelengths of 330 and 350 nm. A plot of the derivative of the intensity ratio at these two wavelengths (330/350) shows a maximum at the unfolding temperature.

### Removing glycosylation

For deglycosylation with NEB PNGase F (New England Biolabs, cat. P0704S), the reaction mixture (250 µL final volume) contained approximately 10 µg of protein sample and 1 mU of PNGase F. Two methods of deglycosylation were used with PNGase F: with and without denaturing the protein. Under the denaturing method, the protein was incubated with 10x Deglyco Denaturing Buffer (containing SDS and DTT) and heated for 10 min, followed by 1 h of incubation with PNGase F and PNGase Glyco Buffer. The non-denaturing deglycosylation was done using PNGase F and PNGase Glyco Buffer at 37 °C for 16 h. The mixture was incubated for 19 h at 37°C. The extent of deglycosylation was examined by SDS-PAGE using Bio-Rad Mini-PROTEAN TGX 4–15% Precast Gels (Bio-Rad, cat. 4561086) with Tris/glycine SDS running buffer (Bio-Rad, cat. 1610732). This step was primarily performed to assess the mass of the protein without glycosylation.

### Mass spectrometry

The mass of all PAP variants was verified by liquid chromatography with mass spectrometry (LC–MS) using an ACQUITY UPLC/VionTM-IMS-QTof system coupled with an electrospray ionization source. Liquid chromatographic separation of samples was performed on ACQUITY UPLC Protein BEH C4 column (300 Å pore diameter, 1.7 μm, 2.1 × 50 mm, Waters) using gradient elution. Then 10 μl of sample was injected with a flow rate of 0.3 ml min^−1^ and the analysis was carried out at default parameters. The acquired data was processed using UNIFI software.

### Mass photometry

Mass photometry measurements were carried out in silicone gaskets (3 mm × 1 mm, GBL103250, Grace Bio-Labs) on microscopy slides that had been cleaned by consecutive sonication in Milli-Q water, isopropanol (Sigma-Aldrich, cat. 33539), and Milli-Q water. 20 μl of 5 nM protein samples were added to the gaskets containing 4 μl buffer and images were acquired for 60 s at 331 Hz. Landing assays were analyzed using DiscoverMP (Refeyn Ltd) to extract particle contrasts.

### Dynamic light scattering

The size distribution of protein in solution was measured by dynamic light scattering using Zetasizer Nano ZS90 (Malvern Panalytical, UK). The sample was measured as it was stored in 0.1M acetate buffer, pH 5.5, and had a concentration of 0.1 mg/mL. The Z-average size (d.nm) and polydispersity index (PDI) were measured and used to characterize the particle size and size distribution in triplicate.

### ELISA

100 uL of various concentrations of proteins were coated in a Corning ELISA 96-well plate (Corning, cat. 3590) for an hour, followed by a wash away of the protein and blocking with 2% BSA (Sigma-Aldrich, cat. A9647) for 1 h. The blocking solution was thoroughly rinsed with PBST, followed by 1 h incubation of 50 µL of i) anti-His (1:10,000) (ab1187), ii) anti-FLAG (1:10,000) (ab49763), and iii) anti-TRAP (1:5,000) (Cat# 1370691, Cambridge Bioscience, UK) in 2% BSA. The antibody was washed with PBST, and the primary antibody reacted against TMB substrate for 50 min until color developed and stopped with an alkaline solution.

## Results and discussion

Candidate mutations selected from the Camsol pipeline, based on the most favorable (negative) predicted ΔΔG (Gibbs free energy change upon mutation from wild type) between –1.3 and –1.6 kcal/mol, identified several high-ranking combinations ranging from one to eight mutations (Table 1**)**. Increasing the number of ‘beneficial’ mutations increased the apparent ΔΔ*G* gain, but > 5 mutations yielded diminishing returns and, importantly, introduced sites with greater risk of charge clustering or catalytic disruption. With five mutations, preferred target residue sites emerged (HIS 22, ALA24, PHE54, HIS197, THR208) avoiding catalytic or glycosylation sites, in flexible or surface regions of the protein, indicating potential for improved thermodynamic stability. Of note, Table 1 shows that the predicted gross ΔΔ*G* is greater than a simple linear addition of those from the individual single mutations. CamSol solubility scores at these preferred sites mostly showed positive values, suggesting that the mutations could reduce aggregation propensity based on sequence and structural context. Thus, taking the balance of both gross scores into account, mutations were selected without perturbing PAP’s catalytic machinery or glycosylation sites and prioritized residues in flexible or surface-exposed regions.

**Table 1:** Comparison of an exemplar selection of mutation groups from CamSol Combination ranked outputs. The selected five-mutation design is shown alongside combinations with fewer or more mutations. Although some larger groups of mutations could achieve higher theoretical scores, these included catalytic, buried, or charge-dense residues (e.g., H203P, A214R, T215K), increasing functional risk and were therefore eliminated by the selection criteria. Smaller groups of mutations predicted less improvement in stability and solubility. The five-mutation flexible or surface-exposed group offered the most robust balance of gain and expected catalytic integrity (selected mutations are in bold)

Mutations included	Number of mutations	ΔΔ*G* (kcal/mol)	ΔCamSol	Feasibility
F54P	1	+ 0.56	+ 0.118	Disrupting an exposed surface hydrophobic patch in a surface loop/turn might disrupt H-bonding with backbone
T208R	1	–1.33	+ 0.114	Lies on the protein surface in proximity to the active-site pocket, but not itself catalytic
A24P	1	–1.50	+ 0.009	Near end of an N-terminal loop
H22R	1	–1.54	+ 0.012	Introduces a permanent positive charge at the surface, away from catalytic metal ligands
H197P	1	–1.56	+ 0.038	In a mobile loop near but not coordinating active-site metal, safe from compromise of the binuclear center
S284P	1	–3.27	+ 0.008	C-terminal, minor solubility effect; limited impact on the folded core
T215K, S284P	2	–4.88	+ 0.115	Loop localized; not sufficient to drive thermal stabilization
V163P, T215K, S284P	3	–6.50	+ 0.181	Good score, adding multiple loop mutations, but includes V163P (buried)
L153P, V163P, T215K, S284P	4	–7.52	+ 0.209	Adds interior residue (L153P); local cluster of mutations
H22R, A24P, F54P, H197P, T208R	**5**	** ~ 8.5**	** + 0.230**	**Spread-out, surface mutations; avoids functional cores**
H28R, A30P, V163P, H203P, T215K, S284P	6	–11.08	+ 0.250	Includes H203P (catalytic); excess charge
H28R, A30P, V163P, H203P, T215K, S284P, A214R	7	–12.11	+ 0.279	Adds A214R (near glycosylation site); charge overload risk
H28R, A30P, L153P, V163P, H203P, A214R, T215K, S284P	8	–13.56	+ 0.310	Maximum score, but includes multiple high-risk sites

**Fig. 1:**
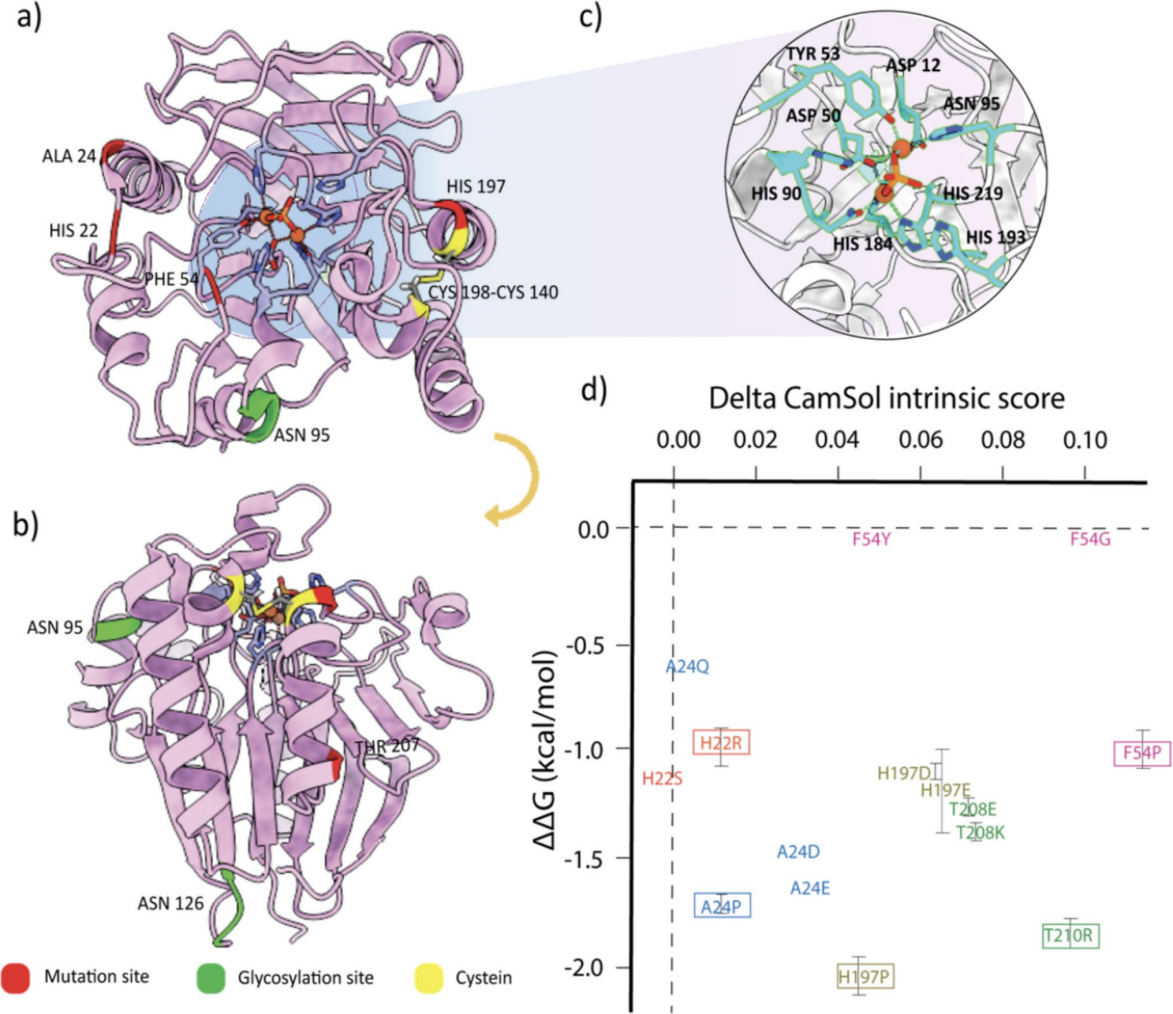
**a–c** **S**tructure of human purple acid phosphatase (PDB: 1WAR), highlighting mutation sites in red and N- glycosylation sites in green. **b** The disulphide bond is in yellow seen in the side view. **c** Focus on the active site of PAP from (**a**), displaying amino acids coordinating with two metal ions and phosphates. **d** Single-mutational scanning results from the CamSol Combination pipeline, displaying the five candidate sites for mutation: HIS 22, ALA 24, PHE 54, HIS 197, and THR208, with their ΔΔG values (y-axis) and difference in CamSol intrinsic solubility (x-axis)

Four of those further selected deliberately avoided highly conserved catalytic or metal-coordinating sites, to preserve activity while improving overall thermostability as detailed further in Table 1** and S1**. Nevertheless, despite being unfavorable in terms of ΔΔG as a single mutation the 5xmutant CamSol output included PHE 54, which is close to the ligand-to-metal charge transfer interaction with Fe(III) (see discussion later). The specific mutations that emerged were HIS 22 to ARG (H22R), ALA 24 to PRO (A24P), to PRO (F54P), HIS 197 to PRO (H197P), and THR 208 to ARG (T208R) (Fig. 1), where mutations with lowest ΔΔ*G* at each site were ranked highest to improve protein stability.

As can be seen from Fig. 1d, while multiple mutations could yield similar ΔΔG values for a given residue, the selected mutations were chosen not only for their favorable computational scores, but also from an understanding of their structural context and expectation of overall thermal stability (Strickler et al. 2006). In this instance, the selection of the individual mutation sites can be rationalized: for example, mutation of ALA 24 (at the start of an α-helix, Fig. 1a, b) to proline, aspartic acid, or glutamic acid all produce a similar ΔΔG (Fig. 1d). Aspartic acid is often known as an alpha helix breaker, but, like glutamic acid, its H-bonding capability and/or formation of salt bridges can stabilize the helix.

Nevertheless, the Suzuki proline rule of thermostability (Watanabe and Suzuki 1997), which anticipates improved stability by replacing an alanine residue with a proline residue in the first turn of an α-helix, is based on decreasing the backbone entropy of the unfolded protein, at the critical start of the helix; this is consistent with the selected A24P mutation rigidifying a flexible N-terminal loop (ΔCamSol + 0.009; ΔΔ*G* –1.50). Similarly, HIS 197, located near an α-helix, adjacent to a cysteine involved in a critical disulfide bond (Fig. 1a, b), was also predicted by CamSol to be advantageous if replaced by proline, aspartic acid, or glutamic acid. This is unsurprising given that residue 197 occupies a structurally similar position to residue 24, where similar substitutions were suggested. Among these, proline had the lowest ΔΔ*G* value (ΔΔ*G* –1.56 ~ 1 kcal/mol lower than the alternatives), likely due to its ability to introduce rigidity into the local structure, potentially stabilizing the environment without compromising the adjacent S–S bond integrity (Ge and Pan 2009). Nevertheless, aspartic acid and glutamic acid could contribute alternative stabilizing effects through hydrogen bonding or electrostatic interactions at this position.

Although the selection criteria for mutations around the metals were designed to exclude residues directly coordinating the metals and phosphate (e.g., TYR 53, ASP 12, ASP 50, HIS 90, HIS 184, HIS 193, ASN 95, HIS 219—see Fig. 1c) and residues within 5 Å of the two metal ions (e.g., GLU 88, ASP 89, HIS 90, HIS 184, TYR 185, HIS 221), phenylalanine residue 54 was not excluded from consideration. Inspection of the 1WAR crystal structure shows that the backbone carbonyl oxygen of Phe54 lies within hydrogen-bonding distance (~ 3.0 Å) to the Nδ1 atom of His90. This interaction likely contributes to stabilizing the conformation of His90, which is a key ligand coordinating both metal ions and the phosphate ion in the active site. It could therefore seem possible that mutating Phe54 to proline would eliminate this backbone hydrogen bond due to the absence of an amide hydrogen in proline. However, although F54P was predicted to be mildly destabilizing in isolation (ΔΔ*G* + 0.56), it provided the largest solubility gain (ΔCamSol + 0.118), targeting a possibly aggregation-prone hydrophobic region. This proline mutation (adjacent to tyrosine residue 53) is in close association with a ligand to metal charge transfer interaction between d Fe(III) and tyrosine-53, with Fe–O–C_tyr_ angle-dependent charge transfer. The Fe–O–C_tyr_ angle is reported as 132^o^ for transferrin and purple acid phosphatase from porcine uterus (Wang 1998; Merkx and Averill 1998; Davis et al. 2002), but any change in the orientation of this coupling, caused by the mutation at the adjacent site, could impact this and potentially influence activity. Nevertheless, increasing local rigidity by the proline (Ge and Pan 2009) in F54P may be advantageous to stabilizing the catalytic activity. The region around the phenylalanine shows poor solubility and introducing proline could mitigate this by disrupting β-sheet formation (Fisher 2006).

The final two mutations introduce the charged amino acid, arginine (pKa 12.48), which increases the net positive charge at physiological pH. In the case of H22R as a single mutation, replacing histidine with arginine showed a ΔCamSol of ~ 0.012 and a ΔΔ*G* of –1.54 kcal/mol. This leads to a higher pKa for the side chain, ensuring that the residue remains positively charged across a wider pH window, thereby reinforcing electrostatic interactions, mitigating a surface aggregation hotspot through increased positive charge. For T208R (ΔCamSol + 0.114; ΔΔ*G* –1.33), the introduction of a charged side chain also enhances surface charge, further promoting salt-bridge formation with GLU 210 at the start of the α-helix and contributing to structural stability (Sazaki et al. 2004; Matsutani et al. 2011) by increasing the helix melt temperature.

In summary, therefore, considering the collective impact of these 5 CamSol predicted mutations, improved thermal stability is anticipated due to α-helix stabilisation at residues 22, 24,197 and 208, changes in pH sensitivity and salt bridge capability due to charged amino acids at residues 22 and 208. Impact on the absorption wavelength for the ligand to metal charge transfer, arising from the mutation at residue 54 influencing the tyr53-to-Fe(III) charge transfer is also a potential outcome and the overall impact of the mutations requires experimental validation.

**Fig. 2:**
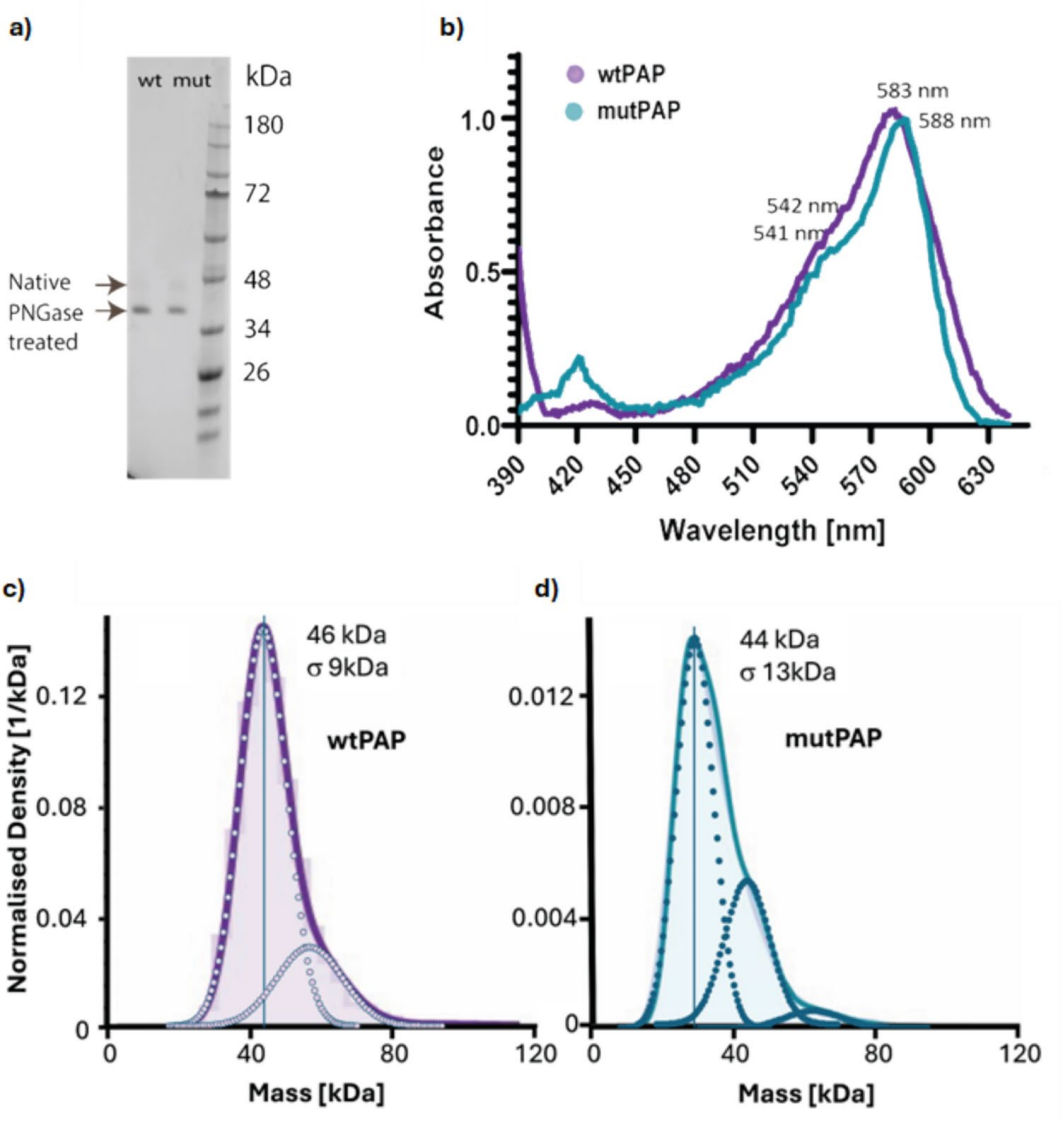
**a** SDS-gel of expression of wt and mut PAP after PNGase treatment and light band indicating glycosylated PAP. **b** UV–Vis showing wtPAP and mutPAP at ~ 580 nm absorbance in the oxidized state and ~ 540 nm in the reduced state seen in mutPAP. **c** and **d** Mass photometer data of glycosylated wt and mut PAP with Gaussian fits to the data asymmetry

In terms of the expressed 5 × mutated PAP protein, ELISA results, using an anti-TRAP antibody targeting the N-terminal of the recombinant protein, confirmed the expression of both wild-type PAP and the mutant (mutPAP) as shown in Figure S1 and their successful expression was confirmed by SDS-PAGE, which revealed bands at ~ 40 kDa and ~ 46 kDa, corresponding to the expected molecular weights of glycosylated PAP (Figure S2). Mass spectrometry analysis of the purified protein showed multiple peaks indicative of glycosylation (Fig. 2 and S2), while deglycosylated samples yielded a single peak at 34.79 ± 0.11 kDa, consistent with the theoretical mass of non-glycosylated PAP (Figure S3). Mass photometry measurements (Fig. 2c,2d snd S4) further demonstrated glycosylation of the proteins, with wild-type PAP at 46 ± 9 kDa (*n* = 3) and mutant PAP giving 44 ± 13 kDa (*n* = 3). An independent *t*-test revealed no statistically significant difference in the overall mass between the glycosylated wild-type and mutant proteins (*t*(4) = 0.45, *p* = 0.68), suggesting that the mutations did not significantly alter the glycosylation pattern of PAP; this is consistent with the fact that the mutations were strategically introduced at positions distal to known glycosylation sites, although the degree of total glycosylation may be affected.

A more detailed look at the mass photometry (Fig. 2c,2d) of the glycosylated wtPAP and mutPAP, when stored in 0.1 M acetate buffer, confirmed the major peak with molecular mass equivalent to a monomeric protein, but with greater variation in the mutant, which may indicate some change in the total glycosylation pattern. However, given the limitations in detecting subtle mass differences in PAP using mass photometry, particularly as the protein's mass approaches the lower detection limit of this technique (Figure S4), this result is not definitive evidence of the glycosylation pattern.

**Fig. 3:**
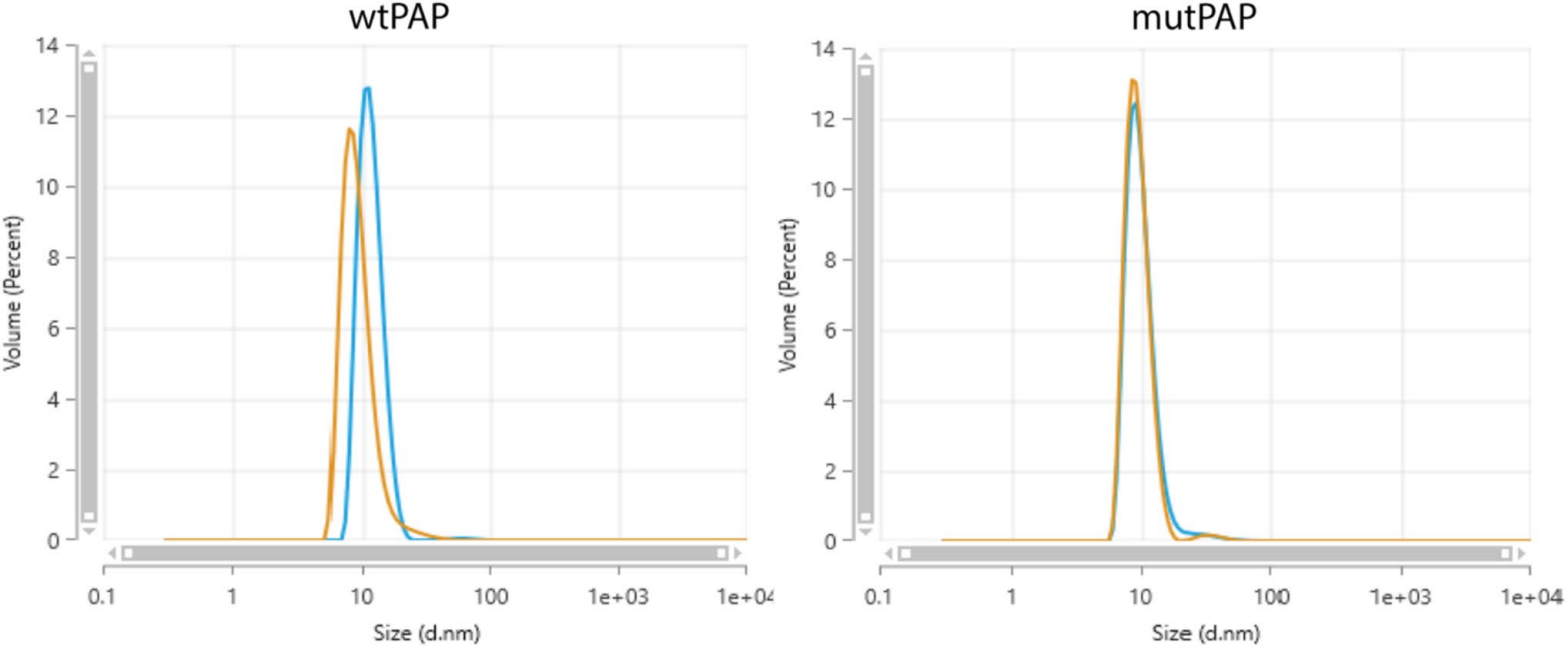
Dynamic light scattering analysis of wtPAP and mutPAP weighted by particle number (blue) and particle volume (orange) in acetate buffer, pH 5.5

Dynamic light scattering (DLS) can be used to assess changes in the solubility of proteins by analyzing their aggregation behavior in solution. Although F54P (ΔCamSol + 0.118) and T208R (ΔCamSol + 0.114) were predicted to provide the largest solubility gains of the mutations, DLS for both wild type and mutant still showed a single peak in the size range of ~ 10 nm (Fig. 3), ruling out aggregation and suggesting, as expected, that glycosylation may play the greater role here in solubility and in preventing dimerization and further aggregation. The coincidence between the number and volume weighted dynamic radius (Fig. 3**)** for the mutant PAP and the closeness of the dynamic ratios for the wild-type PAP suggests a relatively monodisperse particle size distribution that has not been significantly influenced by the mutations. Furthermore, even though the DLS after 6 months storage (Figure S5) indicates that particle distribution is less homogeneous for the mutant, the size difference is very small, not suggestive of reduced solubility and more consistent with time-dependent changes in glycosylation pattern than protein aggregation.

Turning to the metal center, the specific coordination environment in PAP, which may involve a bridging ligand or variations in coordination saturation and geometry, can modulate the energy required for electron transitions between different orbitals. Proton-coupled electron transfer (PCET) from tyrosine to the Fe-center is further influenced by water hydrogen bonding in the active site and local pH changes (Nilsen-Moe et al. 2024). The characteristic purple/pink color of PAP enzymes is predominantly due to the low-energy ligand-to-metal charge transfer (LMCT) band, where the tyrosinate (deprotonated tyrosine) donates an electron to the ferric ion (Nilsen-Moe et al. 2020). This interaction influences the electron density and optical properties of the enzyme. Given that PHE 54 is mutated to PRO, a rigid amino acid, and is adjacent to TYR 53, which participates in LMCT with the binding site, this mutation could have a subtle impact on the peak absorbance of the protein in both its isolated (partially reduced) and fully oxidized states. Wild-type purple acid phosphatase (PAP) exhibits a characteristic pink or purple hue that is more intense compared to its mutant variant at equivalent concentrations. This coloration in PAP results from coupled high-spin iron: in the reduced form as Fe(III)–Fe(II) and as antiferromagnetically spin-coupled Fe(III)–Fe(III) in the oxidized form. The color that arises from a tyrosine-to-Fe(III) charge transfer transition is typically observed between 560 and 590 nm. In the wild type, the absorbance peak is noted at 583 nm (purple), while in the mutant it is red shifted to 588 nm (purple) (Figure 2b). If there are changes in the Fe–O–C_tyr_ angle caused by the F54P mutation, it will alter the ligand to metal charge transfer, since transitions are possible from both the axial or equatorial lone pairs on the tyrosinate to the d-orbitals on the Fe. Depending on alignment, bonding with more or less *π*- or pseudo-*σ* character will result. The 5 nm red shift in absorption wavelength suggests a lower energy in the catalytically inactive Fe(III)–Fe(III) oxidized mutant variant compared with the wild type. The active reduced Fe(III)–Fe(II) PAP has previously been observed in the literature (Merkx and Averill 1998) at a lower wavelength, and in contrast to the oxidized PAP, there is little change from the wild-type peak at 542 nm (pink), to the mutant PAP peak at 541 nm (pink) reported here.

**Fig. 4:**
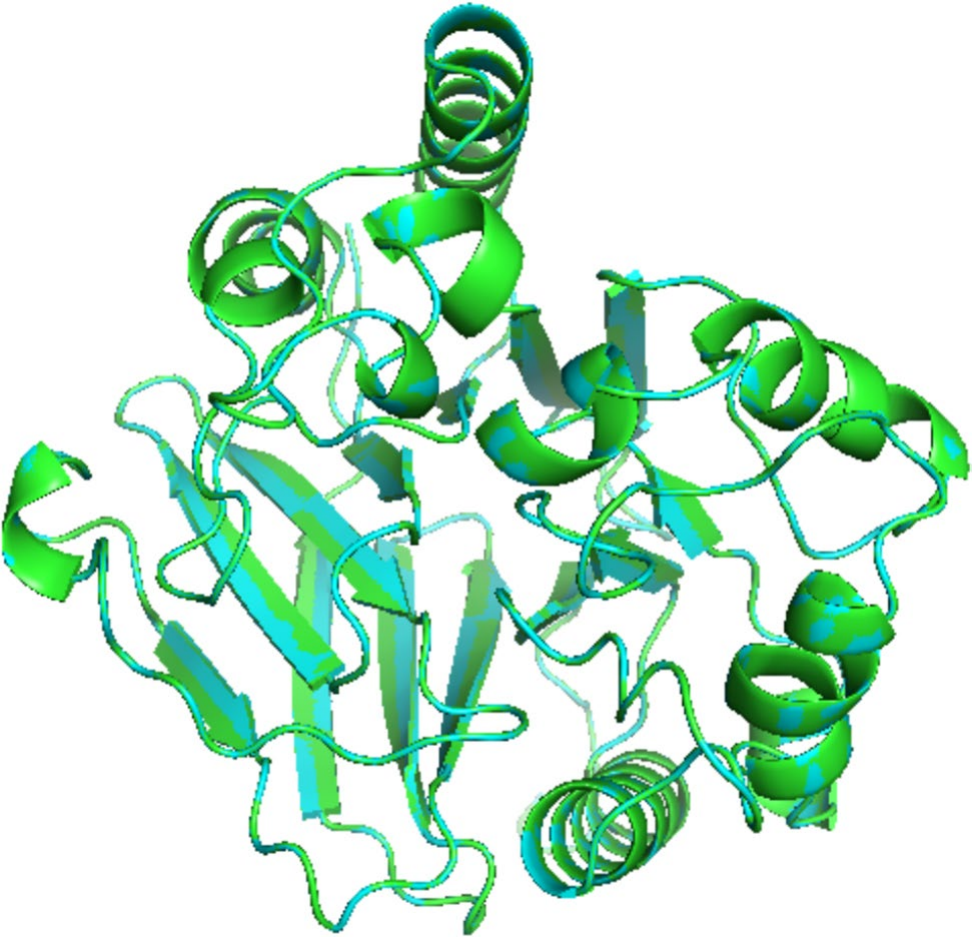
Comparision of wtPAP and AlphaFold-generated mutPAP: superposition of AlphaFold v2.3.1-generated models for wild-type (blue) and 5 × mutant PAP (green) reveals no significant backbone deviations. Local pLDDT scores at the mutation sites (H22R, A24P, F54P, H197P, T208R) were ≥ 92, indicating very high confidence in the structural predictions

To try and further investigate whether these small wavelength shifts could reflect some structural change in the mutant, the respective AlphaFold structures were generated for both the wild-type and mutant PAP. Examination of the AlphaFold output showed consistently high pLDDT (per-residue measure of local confidence) scores (> 80) across the entire structure, including regions adjacent to all five mutation sites. For the specific mutations introduced (H22R, A24P, F54P, H197P, and T208R), local pLDDT scores were ≥ 92, indicating very high confidence in the predicted local structure. The resulting models revealed no significant differences in the overall fold or backbone conformation between the two variants (Fig. 4). Since all five of the selected mutations lie on surface-exposed loop regions that are evolutionarily variable, this consistency is reasonable, because AlphaFold is trained to predict the global native fold of proteins from sequence, and tends to produce high-confidence structural predictions, even for single-point or moderate mutations, especially when the mutation sites are not in functionally or structurally critical core regions and unlikely to drastically affect global folding.

**Fig. 5:**
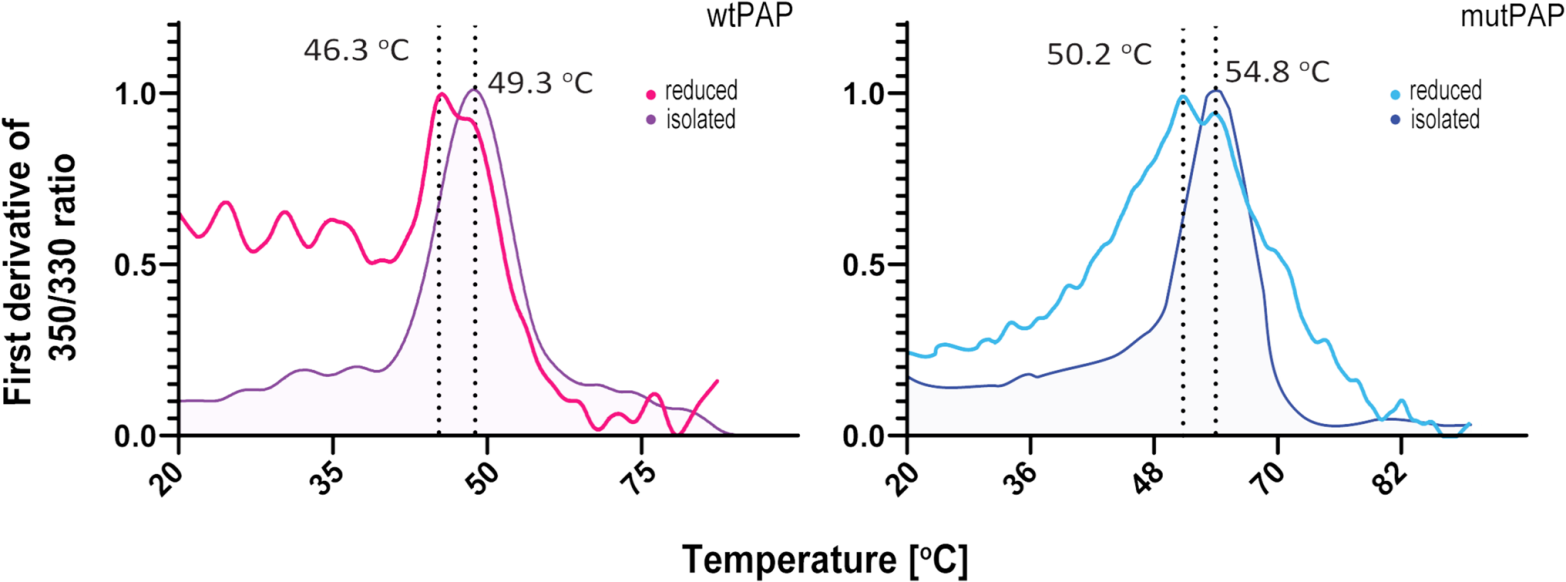
Comparison of thermal stability of wild-type and mutant PAP: thermal unfolding profiles for isolated and reduced forms of wild-type and mutant PAP were measured using nano differential scanning fluorimetry. The first derivative of the 350 nm/330 nm fluorescence ratio is plotted during a temperature ramp at 1 °C/min. The inflection point of each curve represents the melting temperature (Tm), defined as the temperature at which half the protein population is unfolded. Mutant PAP displays a higher Tm compared to wild type, indicating enhanced thermal stability

To assess protein stability, thermal unfolding profiles were obtained, as shown in Fig. 5. The unfolding inflection points, indicating the temperatures at which unfolding initiates, were identified at 49.3 °C for the wild-type protein and 54.81 °C for the mutant in their oxidized form and 46.3 °C for the wild-type protein and 50.2 °C for the mutant in their reduced form. This increase in thermal stability suggests that the mutations do contribute to a more thermostable protein structure.

The activity of wtPAP and mutPAP , measured with pNPP at different pHs produced similar *K*_cat_ values between wtPAP and mutPAP across all pHs: K_cat_ (t = 0.12, *p* = 0.91) or *K*_M_ (*t* = − 0.038, *p* = 0.97) comparing wtPAP and mutPAP, indicating similar catalytic activity and substrate affinity trends (Table 2) (*p* > 0.05) between the two enzymes.

**Table 2:** Catalytic parameters (*k*_cat_ and *K*_M_) of wild-type and mutant PAP across pH 4.0–8.5. Michaelis–Menten parameters were determined using p-nitrophenyl phosphate (pNPP) as substrate, based on absorbance at 410 nm. Assays were conducted at 32 °C across a range of pH values to determine *k*_cat_ and *K*_M_ for wild-type and mutant PAP

pH	wtPAP *k*_cat_ (min^−1^)	wtPAP *K*_M_ (μM)	mutPAP *k*_cat_ (min^−1^)	mutPAP *K*_M_ (μM)
4.0	166 ± 9	885 ± 77	1086 ± 10	951 ± 84
4.5	15,950 ± 1637	1609 ± 233	8380 ± 360	1450 ± 125
5.0	126,980.66 ± 15,772	1183 ± 145	132,499 ± 20,816	1056 ± 54
5.5	191,110.66 ± 12,620	986 ± 108	106,362 ± 15,266	2000 ± 193
6.0	10,898.33 ± 2081	900 ± 58	63,825 ± 9007	929 ± 73
6.5	1966.33 ± 450	2273 ± 143	1353 ± 105	1401 ± 99
7.5	1333.33 ± 152	1609 ± 81	3122 ± 344	1522 ± 103
8.5	67 ± 15	2288 ± 312	862 ± 35	2509 ± 171

**Fig. 6:**
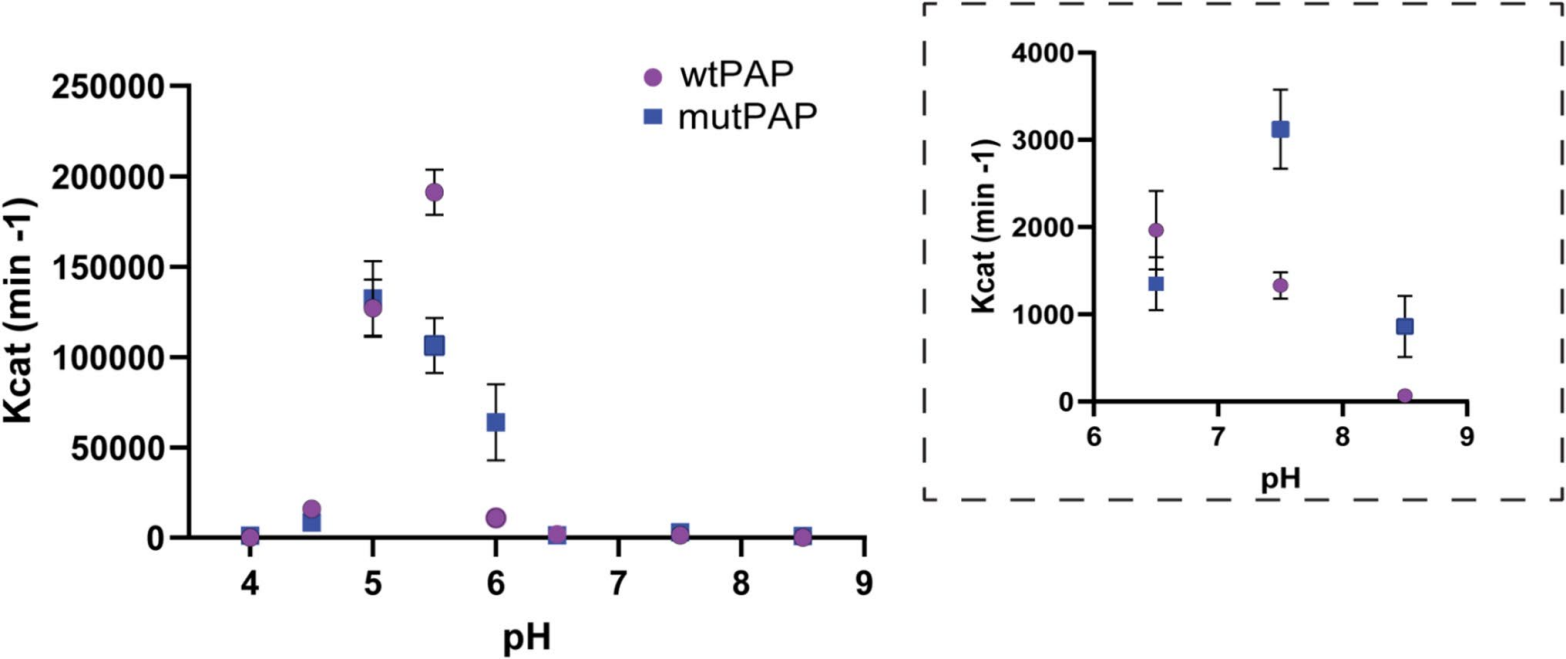
Activity measured as *k*_cat_ for mutPAP and wtPAP from pH 4 to 8.5

For both enzymes, optimal activity is observed around pH 5–5.5 and at 32 °C, where k_cat_​ values peak. wtPAP exhibits its highest measured *k*_cat_​ of 191,111 min^−1^ at pH 5.5, with a corresponding *K*_M_​ of 986 µM, and mutPAP shows its highest *k*_cat_​​ of 132,499 min^−1^ at pH 5, with a similar *K*_M_ of 1,056 µM. Hence, there is a shift in optimum pH with ~30% reduction in *k*_cat_, but comparing just the optimum wild type, pH 5.5, mutPAP exhibits a ~44% decrease in *k*_cat_ compared to wtPAP. This suggests that while the mutation does not impair activity at all pH levels, it may influence catalytic efficiency around the optimal pH. At the extreme pH levels measured (4.0 and 8.5), both enzymes show very reduced *k*_cat_​​ (<1%), indicative of diminished catalytic efficiency, although mutPAP has slightly higher residual activity. Substrate affinity, as seen in *K*_M,_ is also similarly affected by pH in both cases: *K*_M_​ increases at high pH values, indicating reduced substrate binding affinity, consistent with an acid phosphatase and the observed lower activity (Fig. 6**).**

As discussed earlier, the mutations were designed to exclude residues directly coordinating the metals and phosphate and affecting the active site, so these differences were not expected to be significant and may be the result of the impact of F54P adjacent to the LMCT and the flexibility of the disulfide bridge close to H197P, as well as the H22R and T208R influencing surface charge, thereby altering pH sensitivity (Bonvin et al. 2015).

## Conclusion

Purple acid phosphatase (PAP) is a highly evolved metalloprotein, whose intricate structure and redox-active metal center make it particularly sensitive to mutations. Even minor alterations can significantly impact its functionality, necessitating careful re-engineering to preserve both activity and stability.

In this study, we demonstrate, for the first time, the successful enhancement of human purple acid phosphatase (PAP) thermal stability through targeted computational design using the CamSol Combination approach. By specifically introducing the mutations H197P, A24P, F54P, H197P, and T208R, we predicted significant improved protein solubility and achieved a notable increase of 5 °C in thermal stability without affecting the enzyme’s essential catalytic function.

Mutations were strategically chosen to avoid critical functional regions, such as the active site and glycosylation sites, and instead targeted physicochemical properties like hydrophobicity and surface charge to stabilize the protein structure. Based on this specification, the computationally predicted and selected mutations were selected to enhance PAP’s thermal stability.

From the CamSol pipeline, small combinations (1–2 mutations) provided limited cumulative gains, insufficient to predict the experimentally observed + 5 °C increase in melting temperature. In contrast, CamSol predicted 5xmutation designs with optimal compromise between computational scoring and biophysical conservatism. This appeared to be a “mutation plateau”—beyond which the addition of further mutations yielded diminishing returns and introduced greater risk of charge clustering or catalytic disruption, often including functionally sensitive residues (e.g., H203P, A24E) or overly charged local regions (e.g., T208K), raising concerns over misfolding, loss of activity, or increased polyreactivity.

Experimental validation confirmed that the 5xmutant PAP retained catalytic activity, but was somewhat reduced compared with the wild type at certain pH values, while exhibiting increased thermal stability and possibly broader pH tolerance, underscoring the potential of the rational design approach.

The ability to produce stable, high-yield recombinant PAP is crucial for advancing our understanding of its evolutionary and structural properties, as well as for its potential applications in research and therapeutics. The challenges associated with expressing metalloproteins like PAP, particularly the incorporation of metal ions and the need for proper folding, highlight that computational tool like CamSol Combination, when combined with rational design principles, can contribute to the engineering of complex enzymes. Furthermore, this challenge is particularly pronounced when mammalian proteins are produced recombinantly in other hosts, as they often depend on specific post-translational modifications, such as glycosylation, for proper folding and function. In expression systems like *E. coli*, which lack the machinery for these modifications, proteins are prone to misfolding and aggregation, often resulting in non-functional conformations that deviate significantly from their native structure (Dill et al. 2008).

Efforts have been made to express PAP in systems like *Pichia pastoris* (Sträter et al. 2005), CHO cells (Wang and Andersson 2007), insect cells (Marshall et al. 1997), and *Spodoptera frugiperda* (sf9) cells, which have led to successful production and crystallization of recombinant PAP (Hayman and Cox 1994). However, attempts to express PAP in *E. coli* have often resulted in insoluble aggregates or inactive protein, likely due to the absence of glycosylation machinery and the prokaryotic system's inability to maintain the redox environment required for the metal center (Merkx and Averill 1998). Thus, improving solubility and stability, without dependency on glycosylation, also remain primary research goals, particularly for recombinant protein production.

The current model of expression in a mammalian system, as used in this study, would also allow analysis of the impact of mutations targeting the glycosylation sites, as well as fine-tuning of the pH profile and absorption–emission properties. By examining the impact of these factors on stability, the results will provide insight for PAP’s future expression as a non-glycosylated protein in *E. coli*. Further optimization of solubility and yield would offer a cost-effective and scalable expression system. Moreover, the framework established in this study, combining computational predictions with experimental validation, can be applied to other proteins, paving the way for the development of novel enzymes with improved stability and functionality for industrial, therapeutic, and research applications. By leveraging CamSol's predictive capabilities, we provide a foundational strategy for engineering robust PAP variants, opening new avenues for their broader applicability in biotechnology, environmental remediation, and potentially therapeutic contexts.

## Supplementary Information

Below is the link to the electronic supplementary material.

Supplementary file1 (DOCX 633 kb)
