# Correction: Modifying recombinant purple acid phosphatase using computational design

**DOI:** 10.1007/s00249-025-01792-6

**Published:** 2025-08-09

**Authors:** Aishwarya Venkatramani, Montader Ali, Olga Predeina, Jennifer C. Molloy, Pietro Sormanni, Elizabeth A. H. Hall

**Affiliations:** 1 Department of Chemical Engineering and Biotechnology, University of Cambridge, Cambridge, CB3 0AS UK; 2 Yusuf Hamied Department of Chemistry, University of Cambridge, Cambridge, UK

**Correction: European Biophysics Journal** 10.1007/s00249-025-01779-3

In this article, the author’s name Aishwarya Venkatramani was incorrectly published as Aishwarya Venkatraman.

The following sentence was missed in the Acknowledgements section:

This project has received funding from the European Union’s Horizon 2020 research and innovation programme under grant agreement No 101004806.

The updated Acknowledgements section should read as given below:

**Acknowledgements** A.V. is supported by the Cambridge Trust and King's College Scholarship. EAHH received funding from UK Research and Innovation (UKRI) Biotechnology and Biological Sciences Research Council (Grant No.1-934333450 BB/W011905/1). P.S. is a Royal Society University Research Fellow (grant no. URF\R1\201461). We acknowledge funding from UK Research and Innovation (UKRI) Engineering and Physical Sciences Research Council (Grant No. EP/X024733/1). M. A. is supported by a Harding Distinguished Postgraduate Scholarsh. This project has received funding from the European Union’s Horizon 2020 research and innovation programme under grant agreement No 101004806.

Now, the original article has been corrected.

