# Supplementary material for: Modifying recombinant purple acid phosphatase using computational design
Source: Eur Biophys J. 2025 Jul 12;55(3):499–511. doi: 10.1007/s00249-025-01779-3 (PMC13319145; doi:10.1007/s00249-025-01779-3)
Supplement: Supplementary file 1 — Supplementary file1 (DOCX 633 kb) [file 249_2025_1779_MOESM1_ESM.docx]

**Modifying Recombinant Purple Acid Phosphatase Using Computational Design**

**Supplementary Information**

Aishwarya Venkatraman*, Montader Ali, Olga Predeina, Jenny Molloy, Pietro Sormanni, Elizabeth A. H. Hall*

**Author information**

Aishwarya Venkatramani

Department of Chemical Engineering and Biotechnology, University of Cambridge, Cambridge, UK
ORCID 0000-0001-9572-9854

Montader Ali

Department of Chemistry, University of Cambridge, Cambridge, UK

ORCID 0009-0004-9022-3896

Olga Predeina

Department of Chemistry, University of Cambridge, Cambridge, UK

ORCID 0009-0007-9146-9208

Jenny Molloy

Department of Chemical Engineering and Biotechnology, University of Cambridge, Cambridge, UK, ORCID 0009-0003-3477-8462

Pietro Sormanni

Department of Chemistry, University of Cambridge, UK

ORCID 0000-0002-6228-2221

Elizabeth A H Hall

Department of Chemical Engineering and Biotechnology, University of Cambridge, Cambridge, UK ORCID 0000-0001-9572-9854

eah16@cam.ac.uk

**Input sequence and Protein sequence:**

wtPAP:
EAEAEFATPALRFVAVGDWGGVPNAPFHTAREMANAKEIARTVQILGADFILSLGDNFYFTGVQDINDKRFQETFEDVFSDRSLRKVPWYVLAGNHDHLGNVSAQIAYSKISKRWNFPSPFYRLHFKIPQTNVSVAIFMLDTVTLCGNSDDFLSQQPERPRDVKLARTQLSWLKKQLAAAREDYVLVAGHYPVWSIAEHGPTHCLVKQLRPLLATYGVTAYLCGHDHNLQYLQDENGVGYVLSGAGNFMDPSKRHQRKVPNGYLRFHYGTEDSLGGFAYVEISSKEMTVTYIEASGKSLFKTRLPRRARPDYKDHDGDYKDHDIDYKDDDDKHHHHHH

mutPAP:

EAEAEFATPALRFVAVGDWGGVPNAPFRTPREMANAKEIARTVQILGADFILSLGDNFYPTGVQDINDKRFQETFEDVFSDRSLRKVPWYVLAGNHDHLGNVSAQIAYSKISKRWNFPSPFYRLHFKIPQTNVSVAIFMLDTVRLCGNSDDFLSQQPERPRDVKLARTQLSWLKKQLAAAREDYVLVAGHYPVWSIAEHGPTPCLVKQLRPLLRTYGVTAYLCGHDHNLQYLQDENGVGYVLSGAGNFMDPSKRHQRKVPNGYLRFHYGTEDSLGGFAYVEISSKEMTVTYIEASGKSLFKTRLPRRARPDYKDHDGDYKDHDIDYKDDDDKHHHHHH


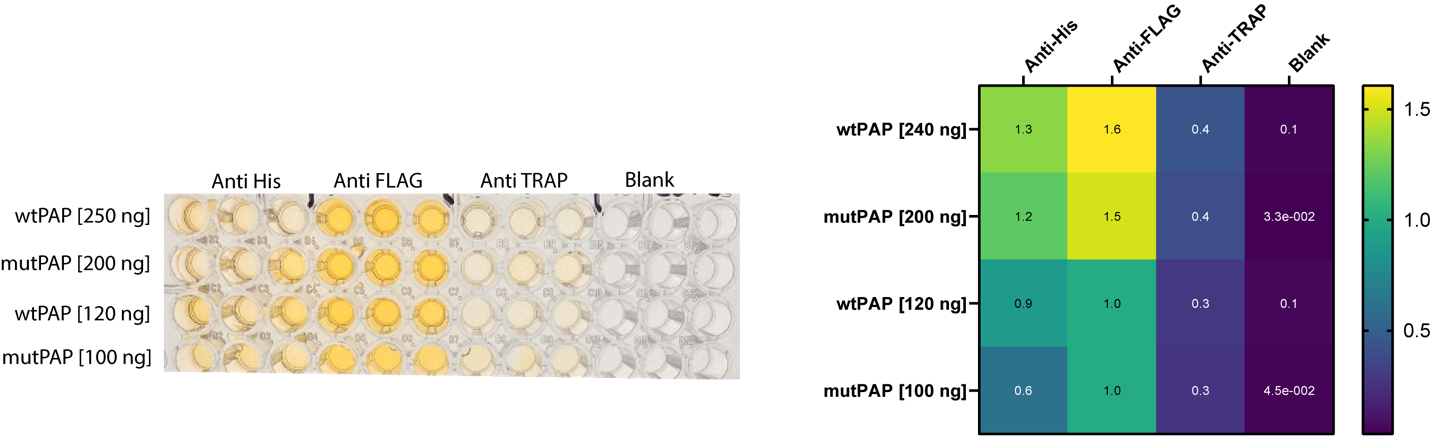


**Figure S1:** ELISA results of wtPAP and mutPAP against HRP conjugated Anti-His, Anti-FLAG and Anti-TRAP.


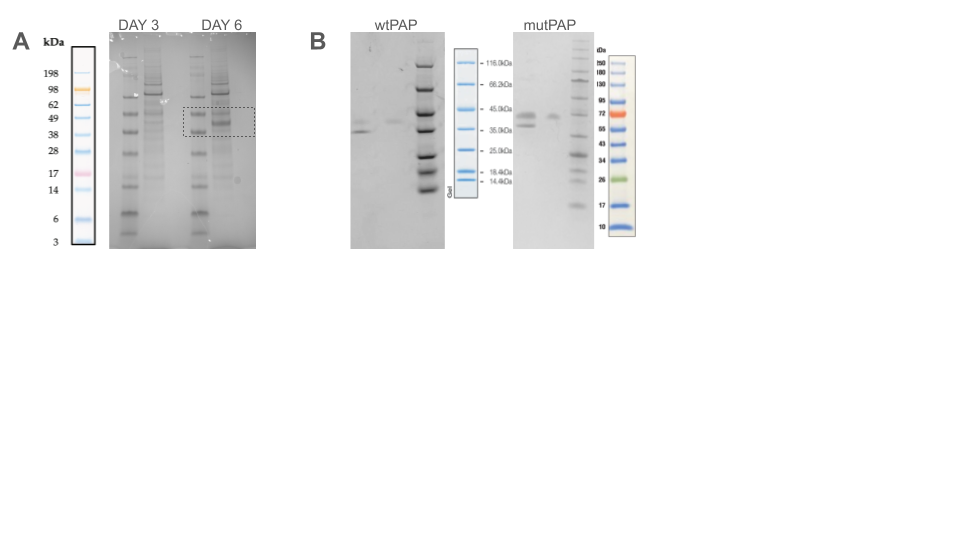


**Figure S2**: (A) Protein expression in Expi293 cells after 3 Days and 6 Days of expression. 6 Days after transfection shows clear expression of PAP sized protein at 45 kDa. (B) PNGase treatment of PAP shows two bands based on the duration of digestion. This figure represents 8 hours of PNGase digestion after 12 hours (left band) and undigested PAP (right). The optimum PNGase digestion condition is for 16 hours as shown in Figure 2 A.


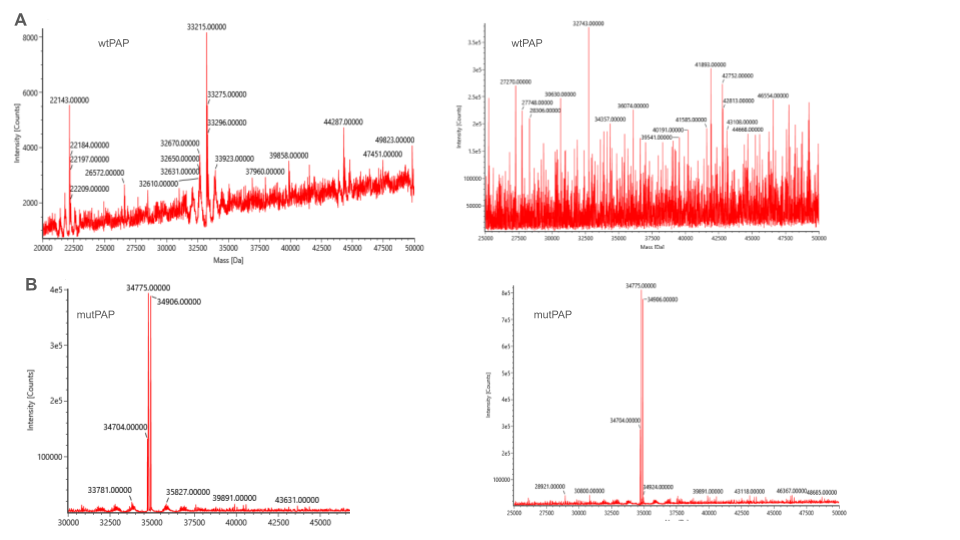


**Figure S3**: Mass-spec of PAP (A) Before PNGase treatment showing glycosylation and inaccurate mass peak and (B) After PNGase treatment + gel filtration. The mass of PAP without glycosylation is found to be 34.79+- 0.11 kDa.

**
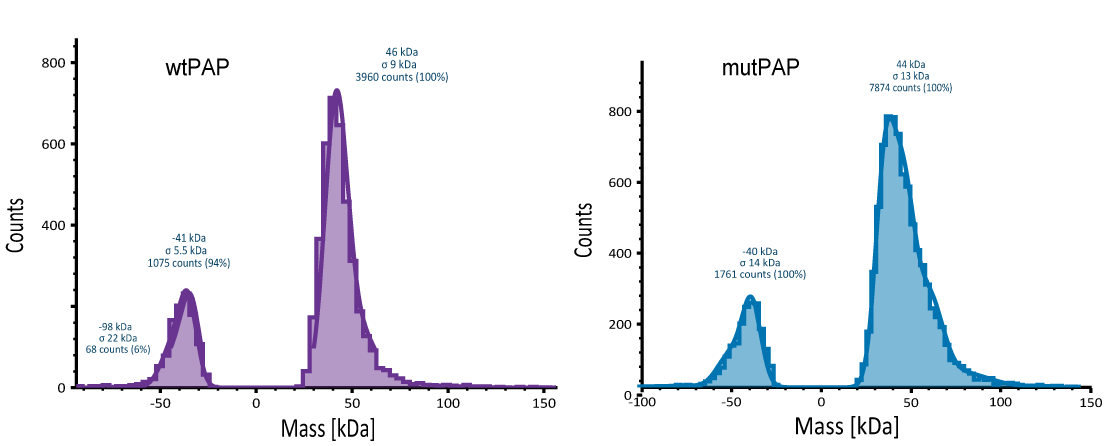
**

**Figure S4**: Mass-photometry reading of wtPAP and mutPAP with noise (left peak)

c


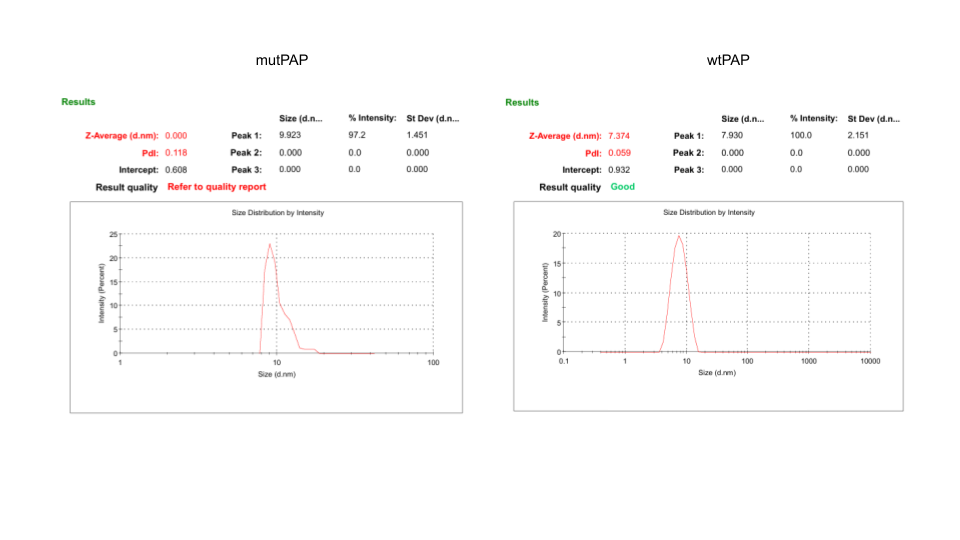


**Figure S5**: Dynamic Light Scattering Analysis of wtPAP and mutPAP after 6 months storage, measured by intensity-weighted size distribution. Peaks for wtPAP (centered ~7.9 nm) and mutPAP (centered~9.2 nm), indicating no significant aggregation.

| **Mutation (WT→Mut)** | **ΔCamSol** | **ΔΔG**  **(kcal/mol)** | **PSSMΔ§** | **Selected?** | **Rationale for Inclusion or Exclusion** |
| --- | --- | --- | --- | --- | --- |
| H22R  His22→Arg | +0.012 | –1.54 | 1.43 | Yes | Included. Large gain in stability; Arg introduces a permanent positive charge at a surface His position (His is partly neutral at physiological pH) to reduce aggregation propensity. Located on the surface, away from catalytic metal ligands, so unlikely to impair function. Predicted to form new salt bridges or H-bonds that stabilize the folded state. |
| A24P  Ala24→Pro | +0.009 | –1.50 | 0.91 | Yes | Included. Proline substitution in an N-terminal loop; significantly stabilizing (rigidifies a flexible region, lowering unfolding entropy) while slightly improving solubility. Situated far from the active site. Chosen to stabilize an order-disorder prone segment at the protein’s edge without affecting catalytic residues. |
| F54P  Phe54→Pro | +0.118 | +0.56 | 0.96 | Yes | Included. Predicted to markedly increase ΔCamSol by disrupting an exposed hydrophobic patch (Phe on the surface). It was mildly destabilizing (+0.6 kcal/mol), possibly due to loss of H-bonding with the backbone, but it was retained because in 5x combination the net ΔΔG is still favorable. Importantly, Phe54 is not in a secondary-structure core (it's in a surface loop/turn), so a Pro can be accommodated without global folding issues. |
| H197P His197→Pro | +0.038 | –1.56 | 1.28 | Yes | Included. Highly stabilizing mutation in a mobile loop near (but not coordinating) the active-site metal. Proline here likely locks the loop conformation, yielding a >1.5 kcal/mol stability gain. This His is not a metal ligand and is semi-exposed, so the mutation does not compromise the binuclear center. Chosen to bolster the local structure surrounding the active site while avoiding any direct active-site residue. |
| T208R Thr208→Arg | +0.114 | –1.33 | 2.63 | Yes | Included. Strongly predicted to improve ΔCamSol and ΔΔG scores. Thr208 (in the wild type) lies on the protein surface (in proximity to the active-site pocket but not itself catalytic). Replacing it with Arg adds a charged side chain that can engage nearby acidic residues or backbone carbonyls, stabilizing the fold. |
| T208K/T208E Thr208→Lys/Glu) | +0.112 (K), +0.095 (E) | –1.27 (K), –1.11 (E) | 2.87 (K), 1.72 (E) | No | Excluded. While both T208K and T208E showed excellent ΔCamSol and ΔΔG improvements, T208R was selected instead due to its stronger PSSM compatibility (PSSMΔ = 2.63 vs. 2.87 for K) and its ability to form broader hydrogen bonding networks via the guanidinium group. Lys is larger and more conformationally flexible, which may introduce unfavorable entropy costs. Glu (a negative charge) was deprioritized because its effect on solubility was slightly lower and its positioning near the already acidic Glu210 might not provide net stabilizing benefit. Hence, T208R was chosen as the most favorable basic substitution at this structurally important position. |
| A24E/A24D Ala24→Glu/Asp | +0.023 (E), +0.014 (D) | –1.08 (E), –0.98 (D) | 0.65 (E), 0.41 (D) | No | Excluded. Acidic substitutions at Ala24 offered reasonable improvements in ΔΔG and minor solubility gain, but were less favorable than the Proline substitution (A24P) ultimately selected. Proline specifically rigidifies the N-terminal loop near the start of a helical region, aligning with the Suzuki rule for helix N-capping. A24E/D introduced charge but lacked structural specificity, and their lower PSSM scores suggested evolutionary disfavor. A24P’s conformational advantage outweighed the modestly better solubility of Glu, leading to its selection for enhanced loop rigidity and helix formation. |
| F54Y/F54G Phe54→Tyr/Gly | ~+0.04–0.05 | Slightly stabilizing (Y), destabilizing (G) | Moderate | No | Excluded. These alternatives to F54P were considered, as F54 lies in a loop flanking an aggregation-prone β region. However, Tyr retains aromatic character, which does not break aggregation potential, and Gly introduces excessive flexibility, which may destabilize folding. Neither variant improved solubility as significantly as Pro, and were therefore rejected in favor of F54P, which, despite mild ΔΔG cost, yielded the strongest anti-aggregation effect. |
| H197D/E His197→Asp/Glu | +0.018–0.022 | ~–1.2 to –1.4 | *High* | No | Excluded. Acidic substitutions at His197 were evaluated as alternatives to Proline. While these mutations improved ΔΔG and added surface polarity, they were excluded due to potential to alter the local hydrogen bond network or disrupt salt-bridge compatibility. Pro197 was selected instead, based on superior predicted ΔΔG and a clearer structural rationale (loop rigidification). |

**Table S1**: Summary of CamSol-Predicted Mutations with ΔΔG Values, Solubility Scores, and Structural Rationales for Mutation Selection in PAP. ΔCamSol is the predicted change in solubility score; positive values indicate improved solubility. ΔΔG is the predicted change in folding Gibbs free energy; negative values suggest stabilizing mutations.
